# Problems in living among breast cancer survivors

**DOI:** 10.25082/CCR.2021.01.005

**Published:** 2021-04-16

**Authors:** Steven S. Coughlin, Deepak Nag Ayyala, Jorge E. Cortes

**Affiliations:** 1 Department of Population Health Sciences, Medical College of Georgia, Augusta University, Augusta, GA, USA; 2 Institute of Public and Preventive Health, Augusta University, Augusta, GA, USA; 3 Department of Medicine, Medical College of Georgia, Augusta University, Augusta, GA, USA; 4 Georgia Cancer Center, Augusta University, Augusta, GA, USA

**Keywords:** breast cancer, cancer survivors, problems

## Abstract

**Purpose:**

Breast cancer survivors may experience worse social, physical, and emotional function compared to the general population, although symptoms often improve over time. Data on problems in living can help to improve interventions and supportive care for breast cancer survivors. Symptoms such as fatigue, pain, difficulties with sleep, and sexual problems may have an adverse effect on the quality of life of breast cancer survivors.

**Methods:**

We examined problems in living using data from a survey of 164 breast cancer survivors who had completed primary therapy for the disease.

**Results:**

A total of 164 women completed the study questions (response rate 16.4%). The mean age of the women was 67 years. Among all participants, 66.7% were white, 29.5% were African-American, and the remainder were of other races. Almost all of the symptoms were more likely to be reported by participants who were < 55 years of age. Other important correlates of symptoms included non-white race, marital status, and having a household income of less than $50,000 per year.

**Conclusion:**

The results of this study highlight the need for caregivers to emphasize screening for and discussion of symptoms, including sleep difficulties, fatigue, loss of strength, aches and pains, and muscle or joint stiffness. Of particular concern are younger survivors and those who are African American or low-income.

## 1 Introduction

In the United States, an estimated 3.8 million women have a history of invasive breast cancer [1]. Improvements in survival rates due to earlier detection and improvements in therapy have led to new challenges related to the quality of survivorship [2]. Previous studies indicate that breast cancer survivors may experience worse social, physical, and emotional function compared to the general population, although symptoms often improve over time [3–5]. Commonly reported symptoms include sleep difficulties, fatigue, pain, cognitive problems, sexual issues, and hot flashes [6]. Such symptoms may have an adverse effect on the quality of life of breast cancer survivors [2]. Frequently, they are due to the therapy received. For example, arthralgias are a frequent side-effect of aromatase inhibitors, which are frequently used for the treatment of postmenopausal women with hormone-receptor-positive breast cancer [6]. Patients who underwent mastectomy or axillary dissection may experience persistent pain at the surgical site or lymphedema in the affected extremity [2]. Data on problems in living can help to raise awareness among patients and physicians, to improve detection, and to design interventions and supportive care for breast cancer survivors.

We thus examined problems in living using data from a survey of 164 breast cancer survivors who had completed primary therapy for their breast cancer. The overall objective was to determine the prevalence and correlates of symptoms and other problems in living.

## 2 Methods

The Cardiovascular Disease outcomes among Breast Cancer Survivors Study (CVDBCS) was a postal survey of a multiethnic cohort of breast cancer survivors who reside in Augusta, GA and who had been treated at Augusta University Health and/or the Georgia Cancer Center. Non-institutionalized women were eligible to take part in the study if they resided in Augusta-Richmond County and Columbia County, GA, or in Aiken County, SC, had been diagnosed with stage I-IV breast cancer, and had completed primary therapy for the disease other than hormonals.

Data were collected using postal survey questionnaires and via abstraction of electronic medical records. The mailings were sent to 1,000 potential research participants who were randomly sampled. A sequential mailing protocol was followed using a modified Dillman method [7]. An advance letter was mailed to the women by the study principal investigator (S.S.C.). The letter provided information about the study (purpose, potential benefits, and risks) and clearly informed patients that they could opt out and not receive further mailings about the study. Three weeks later, a survey consent letter was mailed to women who had not opted out along with a copy of the survey questionnaire and a pre-addressed, stamped return envelope. Women who had not opted out but had not returned a completed questionnaire after 4 weeks of the initial mailing were sent a reminder postcard. Survey responses were checked for completeness and then coded and entered into an electronic database. Questions about demographic factors and breast cancer diagnosis were obtained from a previous study of breast cancer survivors. Respondents were asked about their physical, psychological, and social functioning using the Cancer Problems in Living Scale (CPILS) [8]. The version of the CPILS used in this study was expanded from 31 items to include 50 CPILS items [9]. All of the CPILS items use a three-point Likert-like response scale, with response options being: 0 = not a problem, 1 = somewhat of a problem, and 2 = a severe problem.

After crosstabulations and exploratory analyses of the survey data were completed, logistic regression methods were used to compare groups of breast cancer survivors who did or did not report individual symptoms according to age, race, marital status, and household income. The dependent variable in these analyses was whether or not the respondent indicated that the symptom was “somewhat of a problem” or “a severe problem.” Ninety-five percent confidence intervals (CIs) were obtained for adjusted odds ratios (ORs). Levels of statistical significance were determined using Wald chi-square tests. The goodness-of-fit of each model was examined using the Log-likelihood ratio test.

Another measure of interest we considered is the total number of problems that participants considered as somewhat of a problem or a severe problem. Across the 40 questions asked, the total number of responses with a value of 1 or 2 were recorded. A multivariate Poisson regression model was then fit to identify the association of this score with various factors such as age, race, household income, marital status, *etc.*

## 3 Results

A total of 164 women completed the study questions (response rate 16.4%). The mean age of the women was 67 years (Table 1). Among all participants, 66.7% were white, 29.5% were African-American, and the remainder were of other races. More than half (58.4%) of the women were insured through Medicare and 29.2% held private insurance. The remainder had Medicaid or were uninsured. With respect to breast cancer stage at diagnosis, 19.8% of the women had ductal carcinoma in situ, 26.8% had stage I disease, 21.0% had stage II disease, 8.9% had stage II disease, and 5.1% had stage IV disease. The mean number of years since diagnosis was 9.4 years. About 54.9% of the women reported receiving chemotherapy and only 4.9% reported biologic/targeted therapy.

Problems in living reported by the study participants are shown in Table 2. Symptoms reported by at least 50% of the participants as “somewhat a problem” or “a severe problem” combined included sleep difficulties (53%), fatigue (56%), loss of strength (59%), forgetfulness (57%), aches and pains (71%), and muscle or joint stiffness (67%). Several symptoms were reported as a severe problem by at least 15% of the participants including fatigue (16%), loss of strength (18%), aches and pains (21%), muscle or joint stiffness (17%), dryness in vagina (15%), less sexual desire (23%), and hot flashes (17%).

Almost all of the symptoms were more likely to be reported by participants who were less than 55 years of age (Table 3). Other important correlates of symptoms included non-white race (Table 4), marital status (Table 5), and having a household income of less than $50,000 per year (Table 6). Compared to those who were not married/living with a partner, participants who were married or living with a partner were more likely to report sexual problems, but less likely to report signs of financial distress (Table 5). Lower income participants were more likely to report having major problems with their health and signs of financial distress (Table 6).

For the total score on problems reported as somewhat of a problem/a severe problem, there was a significant relationship between the total score on issues reported as somewhat/severe problem and age (decreasing with age), race (higher for non-whites *vs.* whites), marital status (higher for married women *vs.* non-married women), total household income (higher for low income families), stage of cancer (higher for stages II, III and IV versus ductal in-situ carcinoma) and treatment type (higher for radiation and biologic/targeted therapy *vs.* no-treatment). Coefficients and p-values for the Poisson regression model are presented in Table 7.

## 4 Discussion

The results of this study indicate that a substantial proportion of breast cancer survivors seen at an academic medical center in the southern United States reported having problems in living such as sleep difficulties, fatigue, loss of strength, aches and pains, and muscle or joint stiffness. Important correlates of having reported symptoms included age, race, marital status, and household income. Madelblatt *et al.* [10] noted that older breast cancer survivors may have a high symptom burden due to comorbidities and aging. However, in the current study, almost all of the symptoms were more likely to be reported by participants who were less than 55 years of age. In a study of problems of living among men and women who were adult cancer survivors, Baker *et al.* [8] found that more problems were reported by younger survivors (ages 18–54 years), women, non-whites, those who were not married, and those with a household income < $20,000 per year. Younger cancer survivors are more likely to still be employed and to have dependent family members [8].

Many cancer survivors identify fatigue as one of the most frequent and distressing cancer-related symptom [11, 12]. In the current study, 56% of the participants reported having fatigue. In a population-based study by Meeske *et al.* [11], 41% of breast cancer survivors who were 2 to 5 years following diagnosis were fatigued. These differences in the frequency of fatigue may be due to differences in study design or patient population. A subset of breast cancer survivors experience moderate to severe symptoms years after cancer treatment has ended [11, 13, 14]. Factors associated with fatigue in breast cancer survivors include pain, sleep problems, physical inactivity, and depression [13–16]. Persistent fatigue following cancer treatment affects survivors’ physical well-being and quality of life [11]. Physical activity has been shown to be an effective non-pharmacologic intervention for fatigue in cancer survivors [11, 17–19].

Previous studies have showed that 24% to 84% of breast cancer patients report persistent pain following cancer treatment [2, 20–24]. In the current study, 71% of participants reported aches and pains, which may be partially due to comorbid conditions such as arthritis. About 67% reported muscle or joint stiffness. Report of pain symptoms has been associated with poorer quality of life among breast cancer patients [2]. Results from cross-sectional and longitudinal studies suggest that younger age, more invasive surgery, adjuvant therapy, and psychosocial factors have a role in the development of chronic pain [25, 26]. In the current study, age < 55 years was associated with tenderness at surgical site [OR = 0.33, 95% CI (0.12, 0.88)].

Caregivers should be aware of the increased frequency of problems in living among breast cancer survivors who are non-white or low-income. In the current study, the majority of the non-white participants were African American. The finding that non-white breast cancer patients were more likely to report certain problems in living highlights the importance of considering the special needs of minority patients in their response to illness [8]. Low-income survivors are particularly vulnerable to experiencing problems in living that are indicators of financial distress including being less able to provide for the financial needs of their family, having difficulty in meeting their medical expenses, having difficulty in obtaining adequate insurance, and not having money for the cost of medical visits.

In the current study, participants who were married or living with a partner were more likely to report sexual problems such as vaginal dryness, pain during sexual intercourse, and having less sexual desire. In prior studies, women who received adjuvant therapy experienced more severe symptoms, including vaginal problems, musculoskeletal pain, and hot flashes [27, 28].

With respect to limitations, misclassification bias is a possibility due to the use of self-reported information. The results of this study may not be generalizable to other populations of breast cancer survivors. However, the sample was diverse by race, socioeconomic factors, and history of breast cancer diagnosis and treatment. A further limitation was the cross-sectional design of the study. In addition, selection bias may have occurred due to the low response rate (16.4%).

Regarding the adequacy of the sample size, we carried out sample size calculations based upon a range of estimates. Assuming an alpha value of 0.05 and power of 0.80, an overall sample size of 136 breast cancer survivors, or 68 survivors per group, would be adequate for a two-tailed test on proportions where P1 = 0.30 and P2 = 0.55. Assuming an alpha value of 0.05 and power of 0.80, an overall sample size of 96 breast cancer survivors, or 48 survivors per group, would be adequate for a two-tailed test on proportions where P1 = 0.30 and P2 = 0.60. Assuming an alpha value of 0.05 and power of 0.80, an overall sample size of 130 breast cancer survivors, or 65 survivors per group, would be adequate for a two-tailed test on proportions where P1 = 0.25 and P2 = 0.50. Assuming an alpha value of 0.05 and power of 0.80, an overall sample size of 94 breast cancer survivors, or 47 survivors per group, would be adequate for a two-tailed test on proportions where P1 = 0.25 and P2 = 0.55. These calculations indicate that the available sample size was adequate to detect clinically significant differences across groups.

Taken overall, the results of this study, when combined with findings from previous reports [2,10], highlight the need for caregivers to emphasize screening for and discussion of symptoms, including sleep difficulties, fatigue, loss of strength, aches and pains, and muscle or joint stiffness. Of particular concern are younger survivors and those who are African American or low-income. As more attention is given to increasing the quality of life of breast cancer survivors, it is important to identify problems in living, which can help establish which problems should be the focus of possible prevention efforts and supportive care.

## Figures and Tables

**Table 1:** Characteristics of study participants (n = 164)

Characteristic	Frequency (%)
**Age** (years) mean (SD) (N = 163)	67 (41.1)
**Race** (N = 156)	
White, Non-Hispanic	104 (66.7)
African American, Non-Hispanic	46 (29.5)
Other	6 (3.9)
**Annual Income** (N = 46)	
<$20,000	17 (10.4)
$20,000-$34,999	17 (10.4)
$35,000-$49,999	17 (10.4)
$50,000-$64,999	14 (8.5)
$65,000-$79,999	8 (4.9)
$80,000 +	38 (23.2)
Missing	53 (32.3)
**Number of people in household** (N = 160)	
1	48 (30.0)
2	83 (51.9)
3 +	29 (18.1)
**Employment status** (N = 163)	
Retired	99 (60.7)
Employed	34 (20.9)
On disability	16 (9.8)
Homemaker	9 (5.5)
Temporarily unemployed	4 (2.5)
**Marital status** (N = 163)	
Married/Partner	84 (51.5)
Single	24 (14.7)
Widowed	32 (19.6)
Separated/Divorced	23 (14.1)
**Education** (N = 157)	
Less than HS	5 (3.2)
HS or equivalent	42 (26.8)
Some college	27 (17.2)
Associate degree	22 (14.0)
Bachelor degree	27 (17.2)
Graduate degree	34 (21.7)
**Health Insurance** (N = 161)	
Medicare	94 (58.4)
Private insurance	47 (29.2)
Other	20 (12.4)
**Perceived general health** (N = 162)	
Excellent	16 (9.9)
Very good	58 (35.8)
Good	59 (36.4)
Fair	24 (14.8)
Poor	5 (3.1)
**Breast cancer stage at diagnosis** (N = 157)	
Ductal carcinoma in situ	31 (19.8)
Stage I	42 (26.8)
Stage II	33 (21.0)
Stage III	14 (8.9)
Stage IV	8 (5.1)
Don’t know	29 (18.5)
**Time since diagnosis** (in years) mean (SD) (N = 155)	9.4 (8.8)
**Type of treatment received**(N = 164)	
None	2 (1.2)
Surgery	161 (98.2)
Radiation	111 (67.7)
Chemotherapy	90 (54.9)
Hormone therapy	74 (45.1)
Biologic/Targeted therapy	8 (4.9)

**Table 2:** Problems in living reported by study participants (n = 164)

Description	Total responses	Not a problem N (%)	Somewhat of a problem N (%)	A severe problem N (%)
Sleep difficulties	156	74(47.44)	64(41.03)	18(11.54)
Fatigue	158	69(43.67)	64(40.51)	25(15.82)
Loss of Strength	157	64(40.76)	65(41.40)	28(17.83)
Preoccupation with being ill	154	106(68.83)	37(24.03)	11(7.14)
Major problems with my health	157	100(63.69)	40(25.48)	17(10.83)
Trouble concentrating	152	92(60.53)	47(30.92)	13(8.55)
Being physically unable to have children	138	128(92.75)	3(2.17)	7(5.07)
Being physically unable to have sexual intercourse	151	110(72.85)	31(20.53)	10(6.62)
Forgetfulness	155	66(42.58)	70(45.16)	19(12.26)
Aches and pains	157	45(28.66)	79(50.32)	33(21.02)
Tenderness at surgical site	157	95(60.51)	51(32.48)	11(7.01)
Muscle or joint stiffness	155	51(32.90)	78(50.32)	26(16.77)
Arm swelling	154	126(81.82)	19(12.34)	9(5.84)
Dryness in vagina	153	81(52.94)	49(32.03)	23(15.03)
Pain during sexual intercourse	144	91(63.19)	35(24.31)	18(12.50)
Less sexual desire	148	79(53.38)	35(23.65)	34(22.97)
Hot flashes	151	80(52.98)	46(30.46)	25(16.56)
Sweating	153	84(54.90)	47(30.72)	22(14.38)
Feeling fearful that my illness will return	155	90(58.06)	47(30.32)	18(11.61)
Fears about the future	154	99(64.29)	37(24.03)	18(11.69)
Having difficulty in making long-term plans	154	117(75.97)	26(16.88)	11(7.14)
Feeling emotionally vulnerable	155	116(74.84)	28(18.06)	11(7.10)
Feeling helpless	157	125(79.62)	25(15.92)	7(4.46)
Feeling angry	156	122(78.21)	27(17.31)	7(4.49)
Feeling dependent	156	116(74.36)	27(17.31)	13(8.33)
Feeling isolated	155	124(80.00)	22(14.19)	9(5.81)
Guilt feelings	155	138(89.03)	11(7.10)	6(3.87)
Feeling sad	156	113(72.44)	37(23.72)	6(3.85)
Feeling anxious	156	99(63.46)	45(28.85)	12(7.69)
Feeling less attractive	154	90(58.44)	49(31.82)	15(9.74)
Feeling less sexually desirable	151	96(63.58)	39(25.83)	16(10.60)
Feeling less feminine	155	114(73.55)	29(18.71)	12(7.74)
Difficulty in returning to former roles (e.g. job, family, friends)	156	125(80.13)	15(9.62)	16(10.26)
Job discrimination	149	142(95.30)	2(1.34)	5(3.36)
Difficulty in pursuing the career of my choice	146	126(86.30)	8(5.48)	12(8.22)
Not being able to change jobs for fear of losing my health insurance	140	129(92.14)	3(2.14)	8(5.71)
Being concerned about infection	152	116(76.32)	23(15.13)	13(8.55)
Being concerned about crowds	152	122(80.26)	19(12.50)	11(7.24)
Being less able to provide for the financial needs of my family	151	116(76.82)	22(14.57)	13(8.61)
Difficulty in meeting my medical expenses	153	108(70.59)	23(15.03)	22(14.38)
Difficulty in obtaining adequate insurance	152	129(84.87)	12(7.89)	11(7.24)
Not being able to get the information I need about cancer	152	141(92.76)	4(2.63)	7(4.61)
Not being able to get information I need to take care of myself after treatment	153	139(90.85)	6(3.92)	8(5.23)
Being treated as different from others	153	137(89.54)	8(5.23)	8(5.23)
Problems with family/children	151	137(90.73)	8(5.30)	6(3.97)
Problems communicating with my spouse or partner	144	120(83.33)	13(9.03)	11(7.64)
No regular doctor or medical provider	153	139(90.85)	7(4.58)	7(4.58)
No transportation to/from medical visits	154	145(94.16)	5(3.25)	4(2.60)
No money for cost of or co-payment for medical visits	154	125(81.17)	16(10.39)	13(8.44)
No money for cost or co-payment for medicine	154	128(83.12)	13(8.44)	13(8.44)

**Table 3:** Differences in problems in living by age

Description	OR (95 % CI) (age > 55 *vs*. <= 54 years)	p-value
Trouble concentrating	0.36 (0.13–0.97)	0.0355
Tenderness at surgical site	0.33 (0.12–0.88)	0.0217
Muscle or joint stiffness	0.25 (0.05–0.9)	0.0314
Arm swelling	0.37 (0.13–1.13)	0.0461
Less sexual desire	0.32 (0.11–0.91)	0.0223
Hot flashes	0.09 (0.02–0.34)	0
Sweating	0.17 (0.05–0.51)	3.00E-04
Feeling fearful that my illness will return	0.19 (0.06–0.54)	5.00E-04
Fears about the future	0.27 (0.1–0.72)	0.0047
Having difficulty in making long-term plans	0.30 (0.11–0.83)	0.0167
Feeling emotionally vulnerable	0.26 (0.1–0.72)	0.0043
Feeling helpless	0.28 (0.1–0.8)	0.0107
Feeling angry	0.20 (0.07–0.56)	8.00E-04
Feeling dependent	0.34 (0.12–0.92)	0.0211
Feeling sad	0.16 (0.06–0.45)	1.00E-04
Feeling less attractive	0.32 (0.11–0.88)	0.0203
Difficulty in pursuing the career of my choice	0.22 (0.07–0.72)	0.0057
Not being able to change jobs for fear of losing my health insurance	0.20 (0.04–0.91)	0.0183
Being less able to provide for the financial needs of my family	0.28 (0.1–0.78)	0.0075
Difficulty in meeting my medical expenses	0.23 (0.08–0.62)	0.0013
No regular doctor or medical provider	0.29 (0.08–1.22)	0.0466

**Table 4:** Differences in problems in living by race

Description	OR (95% CI) (non-whites *vs*. Whites)	P-value
Major problems with my health	2.34(1.12–4.95)	0.0213
Tenderness at surgical site	3.45(1.64–7.4)	5.00E-04
Arm swelling	2.83(1.13–7.2)	0.0153
Guilt feelings	4.4(1.38–15.53)	0.0055
Difficulty in pursuing the career of my choice	3.4(1.17–10.44)	0.0203
Being less able to provide for the financial needs of my family	3.91(1.66–9.42)	8.00E-04
Difficulty in meeting my medical expenses	3.23(1.46–7.22)	0.0021
Difficulty in obtaining adequate insurance	6.48(2.28–20.38)	1.00E-04
Not being able to get the information I need about cancer	11.45(2.23–113.57)	6.00E-04
Problems communicating with my spouse or partner	2.7(1–7.34)	0.0311
No regular doctor or medical provider	6.48(1.74–30.07)	0.0016
No transportation to/from medical visits	4.64(0.94–30.02)	0.0307
No money for cost of or co-payment for medical visits	4.03(1.62–10.37)	0.0015
No money for cost or co-payment for medicine	4.51(1.73–12.31)	9.00E-04

**Table 5:** Differences in problems in living by marital status

Description	OR (95% CI) (single or divorced *vs*. married/partner)	P-value
Dryness in vagina	2.28(1.14–4.67)	0.0146
Pain during sexual intercourse	4.62(2.05–11.03)	1.00E-04
Less sexual desire	3.49(1.68–7.45)	4.00E-04
Being less able to provide for the financial needs of my family	0.34(0.14–0.79)	0.0068
No money for cost of or co-payment for medical visits	0.27(0.1–0.69)	0.0034
No money for cost or co-payment for medicine	0.27(0.09–0.72)	0.0047

**Table 6:** Differences in problems in living by household income

Description	OR (95% CI) (income > $50,000 *vs*. < $50,000/year)	P-value
Major problems with my health	0.26(0.12–0.56)	2.00E-04
Being less able to provide for the financial needs of my family	0.38(0.16–0.91)	0.0196
Difficulty in meeting my medical expenses	0.42(0.19–0.95)	0.0306
Difficulty in obtaining adequate insurance	0.35(0.12–0.98)	0.0397
No money for cost of or co-payment for medical visits	0.32(0.13–0.82)	0.0104

**Table 7:** Coefficients obtained from fitting a Poisson regression model to the total number of problems where the participants responded as having somewhat of a problem/a severe problem

Variable	Coefficient	Standard error	P-value
Intercept	3.346388	0.348489	**< 0.0001**
Age	−0.02508	0.002713	**< 0.0001**
Race - Non-whites	0.205097	0.054332	**0.00016**
Marital status - Married	0.25931	0.053476	**1.24E-06**
Income - < $50,000/year	0.352313	0.058528	**1.75E-09**
Medicare	0.158761	0.238918	0.50637
Medicaid	0.432444	0.254269	0.08899
Private insurance	0.266349	0.244818	0.27662
Other insurance	0.02162	0.254306	0.93225
Stage I	0.057097	0.072323	0.42984
Stage II	0.312948	0.075704	**3.57E-05**
Stage III	0.276776	0.099811	**0.00555**
Stage IV	0.355998	0.109878	**0.0012**
Stage - Do not know	−0.020983	0.084564	0.80403
Time since diagnosis	0.005598	0.003226	0.08265
Radiation	0.235654	0.057858	**4.64E-05**
Chemotherapy	0.002194	0.055726	0.96859
Hormone therapy	−0.031402	0.052014	0.54603
Targeted therapy	−0.275192	0.118568	**0.02029**
